# Literary Note

**Published:** 1905-06

**Authors:** 


### LITERARY NOTE.

SURGERY, Gynecology and Obstetrics is the name of a new international magazine to be published monthly at Chicago, under the control of the following editorial staff: John B. Murphy, E. Wyllys Andrews, Frederic A. Besley, Nicholas Senn, J. Clarence Webster, E. C. Dudley, John C. Hollister, William R. Cubbins, Rudolph W. Holmes, C. S. Bacon, Cecil V. Bachellé, Franklin H. Martin, managing editor, Allen B. Kanavel, associate editor.

The first number is announced to appear in July, 1905, and the annual subscription price is $5. We are gratified to note, according to the published announcement, that "the business management is determined to make it a magazine which as a product of illustrated book making will be chaste;"—which we suppose means that the "business management" will censor the photographs and drawings furnished by the authors. The entire circular of announcement bespeaks a high ideal which any journal might be proud to attain.

